# Troubleshooting Interstim Sacral Neuromodulation Generators to Recover Function

**DOI:** 10.1007/s11934-018-0837-5

**Published:** 2018-08-20

**Authors:** C. R. Powell

**Affiliations:** Department of Urology, Indiana University School of Medicine, 535 Barnhill Dr., Suite 420, Indianapolis, IN 46202 USA

**Keywords:** Neuromodulation, Urinary incontinence, Urinary urgency, Urinary frequency, Bladder, Prosthesis and implants, Electric stimulation, Equipment safety

## Abstract

**Purpose of Review:**

Sacral neuromodulation (SNM) is being used to treat lower urinary tract symptoms (LUTS) with growing popularity among clinicians in multiple specialties. As this therapy becomes more common in the USA and Europe, urologists will encounter more patients implanted with SNM generators.

**Recent Findings:**

Over time, it has recently been understood that up to 53% will develop pain at the implant site as reported by Groen et al. (J Urol 186:954, 2011) and 3–38% will lose effective stimulation as reported by Al-zahrani et al. (J Urol 185:981, 2011) and White et al. (Urology 73:731, 2009). There is a paucity of troubleshooting methodology in the literature, apart from revision surgery, to salvage the SNM generator. In fact, it has been suggested that one contemporary series’ failure rate is lower than some historic series because of the ability to reprogram devices as reported by Siegel et al. (J Urol 199:229, 2018). Standard algorithms for such reprogramming efforts are lacking in the literature and may salvage some patients otherwise destined for surgical revision or addition of multimodal therapy to achieve acceptable symptom control.

**Summary:**

It is possible to troubleshoot and thereby salvage many SNM generators, saving patients from surgical revision in many cases and increasing the number of patients with persistent benefit from SNM. The algorithms presented in this manuscript represent a systematic strategy for reprogramming and troubleshooting SNM generators.

## Introduction

Urinary urgency, frequency, urge urinary incontinence, and nocturia, collectively known as lower urinary tract symptoms (LUTS) or overactive bladder (OAB), create significant morbidity for patients in the USA and worldwide [1–3]. First-line therapy includes behavior and dietary modification, while second-line therapy includes medication. Unfortunately, while medication can achieve roughly 70% initial success rates, compliance with medication for OAB is approached 18% after 1 year of therapy [4, 5]. When medication fails to improve symptoms of OAB, sacral neuromodulation (SNM) can be offered as third-line therapy as currently recommended by the American Urologic Association (AUA) guidelines on overactive bladder [4]. A contemporary prospective registry known as the InSite trial has demonstrated long-term safety and efficacy for SNM in light of recent improvements to hardware and technique [6••]. This carefully controlled, carefully studied cohort of patients exhibited undesired changes in therapeutic stimulation despite initial success. Twenty-two percent of patients reported an undesirable change in stimulation following successful implantation, implant site pain in 15%, and ineffective stimulation in 13% [6••]. Many others have reported similar changes in stimulation efficacy, some of these appear in Table 1. As the number of SNM implants worldwide continue to climb, addressing these unwanted changes will become more and more important for clinicians treating LUTS/OAB.

**Table 1:** Contemporary sacral neuromodulation series (SNM) demonstrating an undesired change in stimulation following successful stage 1 trial. Series having abdominal generators were excluded due to the increased rate of revision and pain complications. NA indicated not available from the manuscript

Author	Year	Reprogramming mentioned in manuscript	Number of patients	Change in stimulation	Lost benefit	Pain from device
Hijaz [7•]	2006	Yes	161	NA	16%	2%
Siegel [6••]	2018	Yes	140	22%	13%	15%
Shih [8]	2013	No	142	NA	18%	6%
Peeters [9]	2014	No	217	NA	22%	6%
Al-Zahrani [10]	2011	No	96	13%	38%	14%
Van Voskuilen [11]	2006	No	149	43%	28%	28%
Groen [12]	2011	No	60	NA	NA	53%
White [13]	2009	No	202	NA	3%	3%

**Fig. 1:**
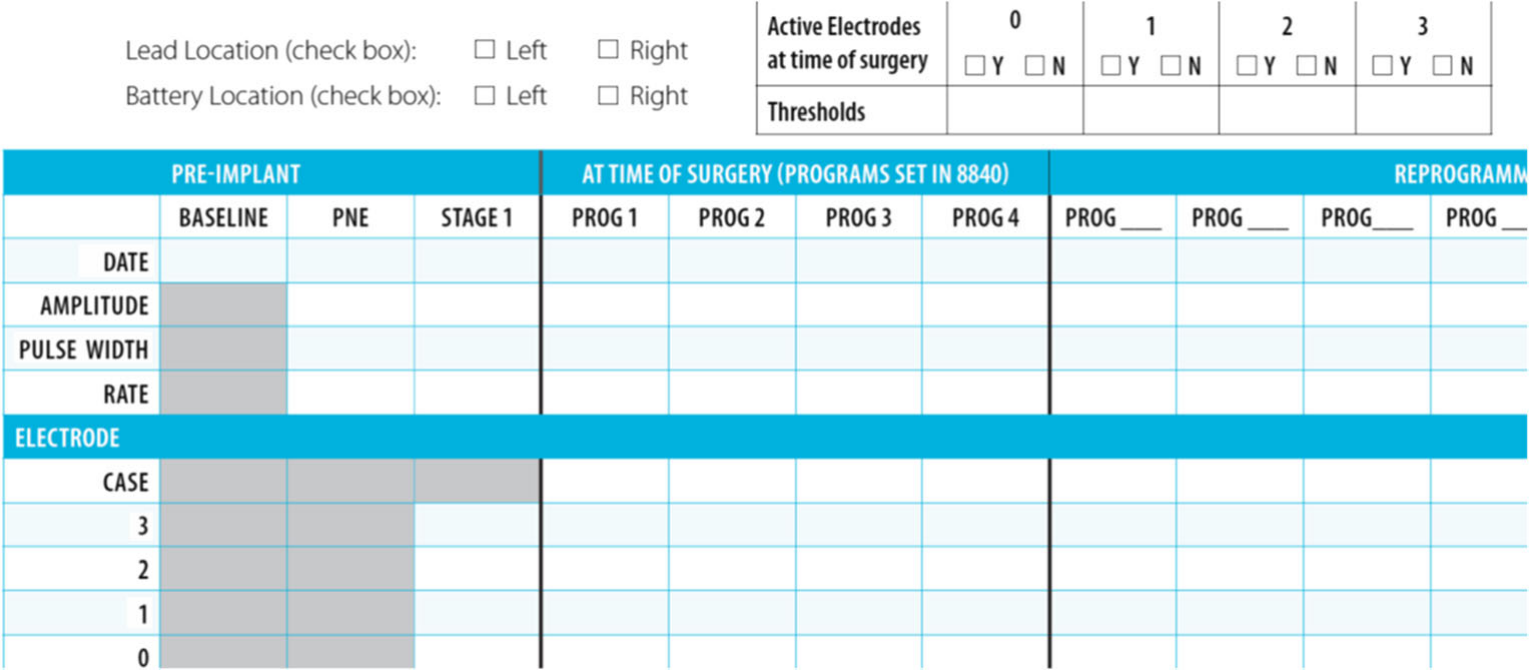
Sample patient worksheet

Many busy practices have found it necessary to troubleshoot these devices following implantation, but standardized troubleshooting algorithms in the literature are rare and may not address all situations [7•, 14, 15•, 16•]. In some cases, physicians rely on representatives of the manufacturer to troubleshoot and reprogram, but in many cases, the most appropriate person to do this is the clinician who implanted the device, since they understand the entire clinical picture, as well as the knowledge of how the lead was implanted and have knowledge of which leads provided the initial response. A step toward standardizing the evaluation process during stage 1 SNM lead placement was taken with the introduction of a standardized Patient Management Worksheet by the manufacturer, Medtronic® (Dublin, Ireland). This contains information about which leads had the best response at the time of implantation, and allows for the recording of lead combinations that have already been tried so this is not duplicated during the trial phase. A modified example can be seen in Fig. 1. Many reviews of SNM have focused on complications related to lead implantation or generator placement. Some have categorized post-implantation problems as infection-related, mechanical problems, and response-related [7•]. Most series consider surgical revision of the lead or generator without mention of reprogramming [17]; however, some articles dedicated to reprogramming do exist [15•, 16•]. The following manuscript will review the types of problems patients may present with following successful stage 2 SNM generator implantation, and some algorithms for programming the SNM generator, as well as proposed causes and solutions for these issues.

The review will be organized by patient complaint, since that is how the problem will be presented to the troubleshooting practitioner, with the intent of providing a clinically useful algorithm to serve as a tool to aid troubleshooters. Articles describing loss of benefit, change in stimulation, and revision rates will be reviewed (Table 1).

It is suggested that regardless of the specific complaint, the clinician does a few things at every encounter:

1. Check the wound for signs of infection or extruded lead or hardware.
2. Obtain a history specifically quantifying urinary frequency, urgency, nocturia, incontinence episodes, and number of pads used, if any. Compare this to past symptoms to assess improvement, worsening, or stability of symptoms. A useful adjunct is a validated questionnaire for LUTS, per the clinician’s preference.

## Patient Complaints

Perhaps the most disappointing clinic experience occurs when a patient who previously was doing well following surgery presents with a problem. Below are some common scenarios patients may present with following SNM implantation.

## Patient No Longer Feels the Stimulation and No Longer Has Good Benefit

This is a frustrating and common phenomenon, especially since the patient previously had a response good enough to justify the generator implant. The first step is to interrogate the device, and this is described in Algorithm 1 below. First, determine that the device is turned ON and the amplitude of stimulation is high enough that a patient can be expected to feel it (i.e., look at the operative report and note at which threshold the motor response was achieved). If the patient still does not feel this level of stimulation, it is necessary to rule out a short circuit or open circuit (crossed wires or broken wires, in layman’s terms). This, too, appears in Algorithm 1. If all appears normal, it is time to change the program (Algorithm 2), and if this is unsuccessful then it may be necessary to alter the SNM signal stimulating the patient’s nerve roots. This process is detailed in Algorithm 3. If none of these measures can restore sensation, then it is necessary to consider a lead migration. A KUB can be a useful study, but the examiner must have obtained a baseline KUB at the time of implantation, and subsequent KUBs must be done with the same precise positioning of the patient. It has been our experience that this is difficult to achieve in most radiology departments, so a telephone call to the technologist can be very helpful in establishing that one is truly comparing apples to apples and not simply a malpositioned film. If lead migration is suspected, then revision of the lead can be carried out. Conservative efforts at reprogramming are advised before lead revision is considered. There have been some cases of electrical interference with the device, particularly with security systems at retail outlets, and particularly with the older 3023 model, and so if the patient reports a history of sudden change in stimulation noted immediately after visiting an establishment using wireless security systems, this should be considered. Often explant of the generator is necessary if a trial of reprogramming fails.

## Patient Feels the Stimulation in the Same Place but No Longer Has Any Benefit

This too is frustrating for the patient, and in addition to the measures described above, it is important to check for common transient causes of LUTS, such as UTI. Algorithm 3 should be considered early in this case, simply reprogramming the pulse width and/or frequency can achieve stimulation in a slightly different location and may achieve benefit. Adding anticholinergics or beta-3 agonists to this treatment plan may provide benefit, as may onabotulinumtoxin A, but clearly patients would prefer a successful outcome from a single modality.

## Patient No Longer Feels the Stimulation but Still Has Good Benefit

In this case, it is often helpful to ensure the SNM generator is on, perform basic SNM generator interrogation (see algorithm 1). As long as the generator is on and stimulation amplitude (the “volts” is not above 5.0 V), the patient can be left alone but with careful follow-up. If the amplitude is elevated, the patient is at risk for early generator depletion and attempts should be made to reduce the stimulation voltage. This can be done by changing the active electrode (Algorithm 2) and seeking to achieve a lower threshold of stimulation. It should be noted that it is not necessary for a patient to feel the stimulation to have a good therapeutic benefit.

## Patient Feels the Stimulation in a Different Place and No Longer Has Any Benefit

This is a similar situation to the lost sensation/lost benefit scenario described above and the same algorithms (1, 2, then 3) should be followed. If it is felt in a different location but not lost, then it is less likely that a broken wire/open circuit will be encountered during Algorithm 1.

## Pain at the Implant Site

This is a common complaint, and during the perioperative period, after surgical site infection is ruled out, can safely be observed in our experience and often resolves. However, if it does not occur in the perioperative period, or does not resolve within a month or two, it may be necessary to consider pocket revision. This complaint was noted in 15% in the contemporary INSITE Seigel series [6••] and up to 29.7% pooling all types of pain in the older-generation SNM series by the same author [18]. Outside of infection, it can be caused by stimulation or by generator malposition. One way to troubleshoot this is to turn off stimulation with the N’Vision Clinician Programmer and have the patient return in a month. If the pain is improved, it may be possible to eliminate the pain with programming alone (Algorithm 3). If not, it may be beneficial to revise the generator pocket. Our experience has been that generators placed directly on gluteus muscle (very thin patients) or patients who have recently lost considerable weight can benefit from pocket revision to get the device to lie parallel to the plane of the skin with some distance from direct contact with gluteus muscle.

## Pain Down the Leg or Foot

Commonly, this suggests a high (2, 3) electrode is stimulating in the S2 region as the lead traverses the nerve root, but it is also possible the lead itself is irritating the nerve root. The first step is to turn off the generator and have the patient return after 2–4 weeks. If turning off the stimulation improves the pain, it may be possible to program out the harmful response by energizing (making NEGATIVE “−”) only electrodes 0 or 1, the most distal electrodes, and routing the return current through the case (making it POSITIVE “+”) as described in Algorithm 2. Another option is to alter the pulse width of the stimulation signal as described in Algorithm 3. Altering pulse width typically changes where the patient perceives the stimulation, and can be used to “move” the sensation from the buttock to anus or genital area by trial and error. If turning off the stimulation fails to improve the pain, it is likely a mechanical or lead-placement issue and a lead revision might be necessary.

## Unwanted Movement of the Leg

This suggests a stimulation of S2 nerve roots and can be programmed out if any of the electrodes traverse the S3 nerve roots. Algorithm 2 is most useful for programming unwanted S2 stimulation out, but if this is unsuccessful, changing pulse width and frequency as described in Algorithm 3 is worth trying. Often, a lead revision is necessary.

## Unwanted Movement of the Foot

Unlike leg movements, foot movements can often be programmed out using Algorithm 2 and Algorithm 3. A strong S3 response can sometimes be frustrating to the patient, especially if the motor threshold is below 1–2 V at the time of implant. Reducing the amplitude of stimulation as well as the frequency can help dampen the discomfort, and ramping up the voltage slowly can also help. Foot cramping can often result with over stimulation of the great toe. Lead revision is an option if multiple reprogramming sessions prove unsuccessful.

## Fever and Tenderness at the Implant Site

Efforts to salvage the infected lead and generator are often unsuccessful, but a trial of antibiotics is not unreasonable, since the morbidity is low and reports of a serious life-threatening infection or sepsis in the literature are extremely rare [7•, 8].

## Transient Electric Shock

Some have proposed that this is likely due to moisture in one of the electrical connections, namely the lead connecting to the generator [14]. It may also be due to subtle movements of the lead and electrodes, which can shift in situ despite fixation with the tined-lead system. Moreover, insulation could be deficient, and although this is unlikely given the thick silicone housing that covers these leads, one should be prepared to consider any possibility when dealing with an implanted device. The first step for this complaint is to turn off the device to determine if the output itself is the cause, or if it is due to a mechanical cause such as the lead putting pressure directly on a nerve root. If turning it off (see Algorithm 1) improves the transient shock, then reprogramming to electrodes furthest from the current settings (Algorithm 2) may help, but it is more likely that a lead revision, without replacing the lead or generator, will help. The goal would be to clean and dry all connections and to tighten all bolts. Although it is highly unlikely that a bolt has become loose or perhaps tightening was overlooked at the time of implantation, all possibilities should be considered. A loose bolt may not appear as an open circuit on Algorithm 1.

## Adverse Change in Bowel Function

Just as an adverse change in urinary function can often be programmed out, it is worthwhile to first follow Algorithm 1 to make sure the SNS generator is turned on, then Algorithm 2 can be used to alter the electrode configuration. CT can be a useful adjunct in this situation because sometimes the distal tip of the lead can be very close to the rectum, and if this is the case, it is worthwhile to start by altering the active electrode so the stimulation is coming from electrode #3 (make #3 NEGATIVE or “−”) and make the case or electrode #2 POSITIVE (“+”) to keep the stimulation away from the rectal wall. Algorithm 3 can be used to alter the signal if sufficient change cannot be achieved by changing the stimulating electrodes. Modulating the stool consistency with fiber or osmotic laxatives such as polyethylene glycol should also be considered, and possibly a consistent bowel program for more severe cases.

The previous paragraphs were organized by symptom, and many of the manuscripts organize adverse responses by presumed cause. Some common causes for the above scenarios are discussed below.

## Lead Migration

Almost every SNM series describes lead migration. After generator implantation, it is reasonable to expect that some patients will exhibit some of the changes and problems described in the preceeding pages. One proposed cause for a change in response is lead migration. This can be suggested by plain KUB, but this is not always definitive. Lead migration has been noted to occur in up to 11.8% by Hijaz and colleagues [14] and 8.4% by Siegel and colleagues [18]. Although this represents a mechanical problem, it can be successfully remedied by reprogramming in some cases, and this should be attempted as described in Algorithm 2 and 3.

## Fractured Lead

Like lead migration, a fractured lead represents a mechanical problem but can often be remedied by reprogramming “around” the broken lead (s). The advantage is that Algorithm 1, impedance testing, can locate the fractured lead pair precisely. This should be suspected when a history of a fall is given. This is very common in children, presumably because they are very active, and in fact Reinberg and colleagues noted a 49% revision rate, even after accounting for and excluding those revised for resolution of symptoms [19].

## Malpositioned Generator

This will often cause pain at the generator site which does not resolve with turning the stimulation off. Moreover, malpositioned generators are often palpable at an unusual angle relative to the surface of the skin. If angled too severely toward the implant scar, they may threaten to erode through the skin. A pocket revision is often necessary, and can most effectively be accomplished by incising the pseudo-capsule surrounding the generator, removing the generator, then incising the back wall of the pseudo-capsule and creating a new pocket parallel to the surface of the skin but deeper than the existing pocket. After the generator is re-implanted, both layers of pseudo-capsule can be closed together to eliminate dead space and reduce the chance of generator migration.

## Conclusion

SNM can be a life-altering therapy for patients suffering from LUTS or OAB but is not without drawbacks. Effectiveness can suddenly decrease following a period of benefit, or it can gradually decline, and clinicians implanting SNM generators can often troubleshoot and salvage these devices.

## Algorithm 1

### The Basics of SNM Generator Interrogation

**Fig. 2:**
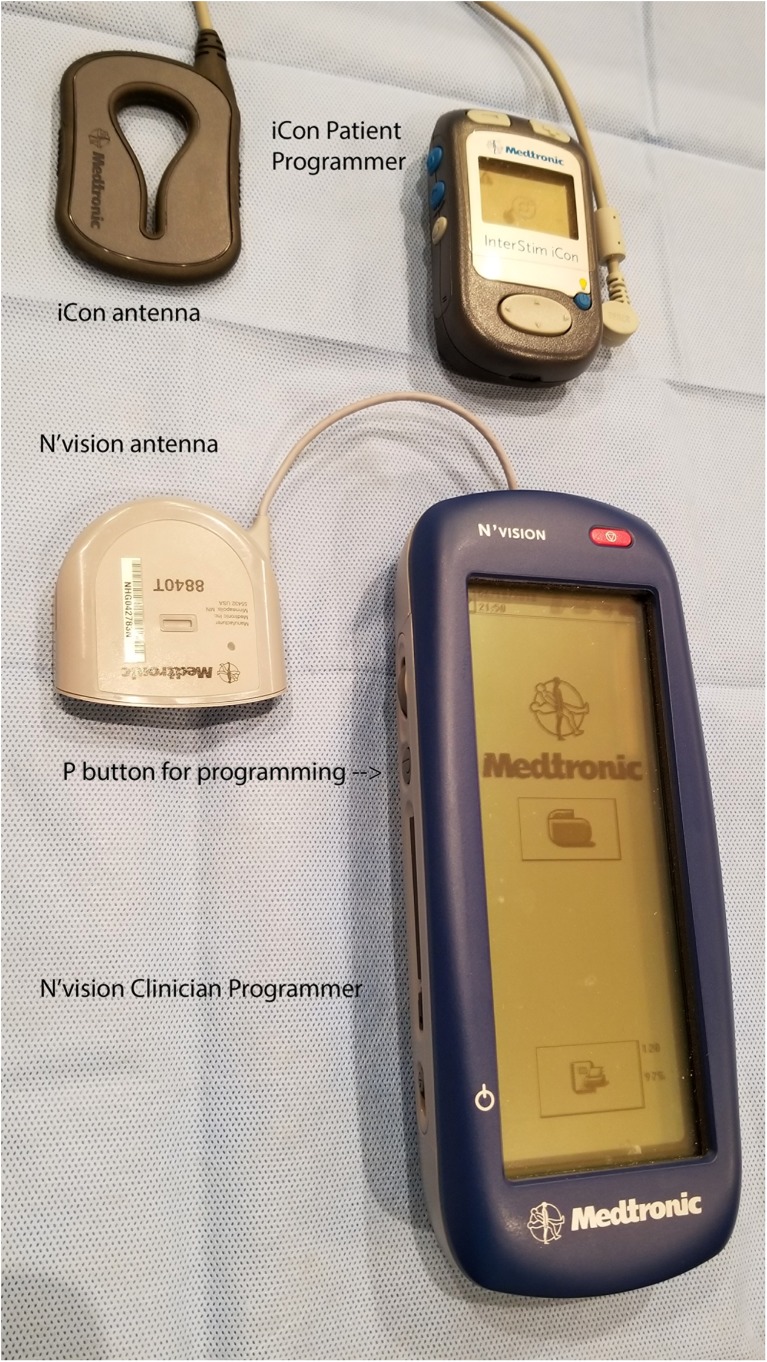
Icon patient programmer and Medtronic Clinician Programmer, with the antenna extended for both. Arrow points to the “SYNC” button of each device. It is necessary to push the “SYNC” button on the clinician programmer to program a SNM device

**Fig. 3:**
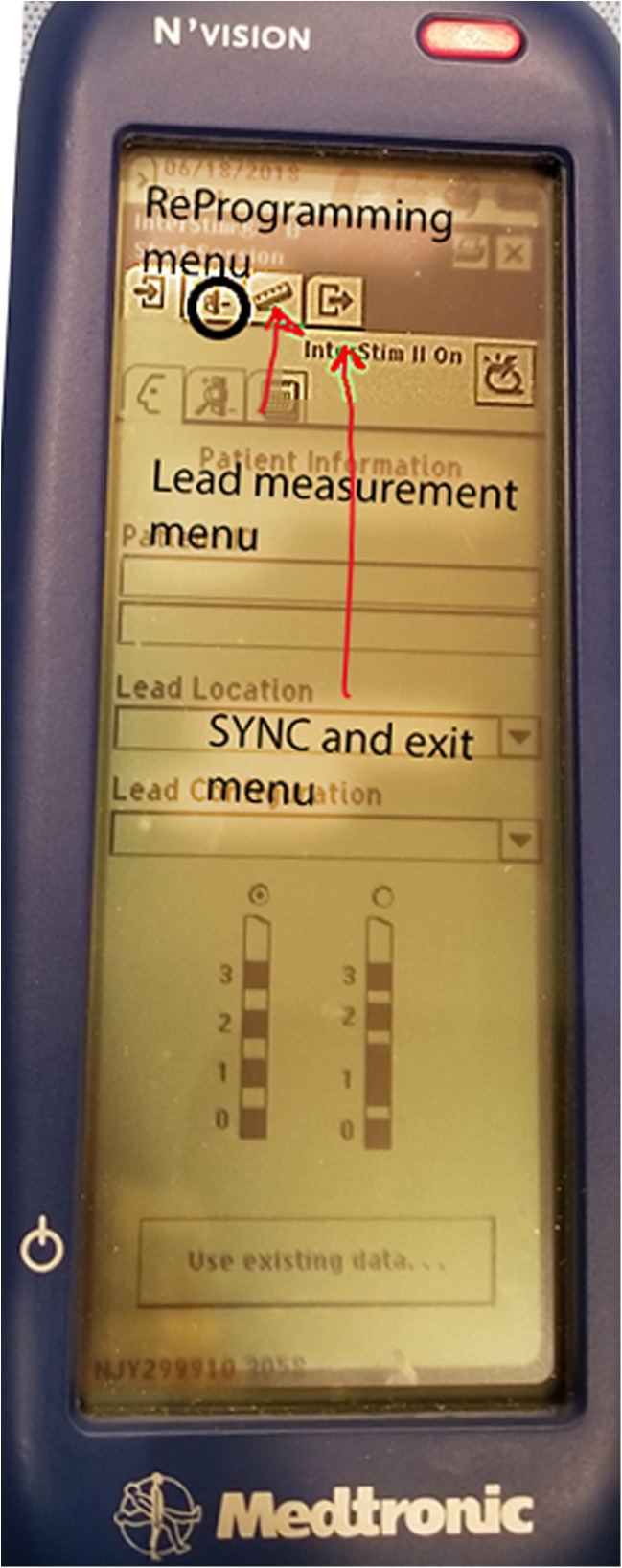
Initial screen demonstrating programs available to the clinician. The top menu is useful for accessing the programs, reprogramming, interrogating lead integrity, syncing the changed programs to the iCon patient programmer, and exiting the programming session. Below the amplitude (“voltage”) indicator, the icon for modifying the stimulation signal itself can be seen

**Fig. 4:**
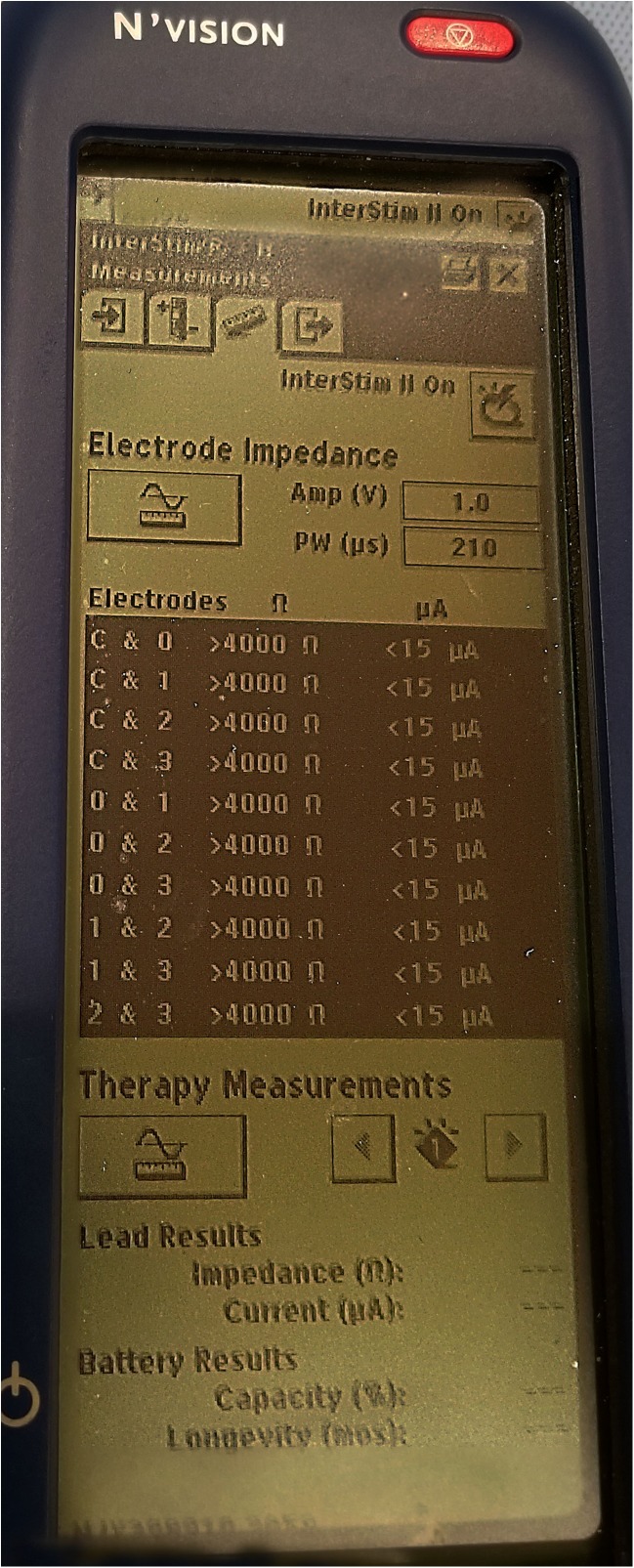
Lead interrogation screen demonstrating broken wires (“> 4000 Ω”) and short circuits, possibly from moisture (“< 50 Ω”)

1. Place the words and LED lighted section of the Clinician Programmer face-up (so they can be seen by the clinician, not the part facing the patient’s skin) and press the SYNC or “P” button (Fig. 2).
2. Once the N’vision clinician programmer has communicated with the SNM generator, sync the iCon Patient programmer (Fig. 2) with the clinician programmer. If it does not connect sometimes it is necessary to cycle the iCon patient programmer on and off using the power switch labeled with the yellow light bulb. Enter the patient’s information if necessary.
3. Make sure the SNM generator has not been turned OFF by looking for the icon on the screen after the N’vision clinician programmer connects with the patient’s SNM generator (Fig. 3).
4. Make sure the amplitude of stimulation is high enough for the patient to feel it
5. Interrogate the leads to ensure there are no open circuits (i.e., Broken wires, designated by > 4000 Ω signal – see Fig. 4) and no short circuits (i.e., Water in the connection or fused wires, designated by < 50 Ω signal – Fig. 4). It is important to note that sometimes a lead pair will return the > 4000 Ω signal yet it is not truly broken. It is thought this is due to increased resistivity of scar tissue that can form around the electrode, diminishing but not eliminating its effectiveness. If this is suspected one can increase the test voltage used to determine open circuits. The process is outlined in Algorithm #4.
6. Use the clinician programmer to change the program and see if the desired stimulation can be achieved

## Algorithm 2

### The Basics for Changing the Program

1. Place the words and LED lighted section of the clinician programmer face-up (so they can be seen by the clinician, not touching the patient’s skin) and press the SYNC button (Fig. 2).
2. Once the clinician programmer has communicated with the SNM generator, sync the iCon Patient programmer (Fig. 2) with the clinician programmer. If it does not connect, sometimes it is necessary to cycle the iCon patient programmer on and off using the power switch labeled with the yellow light bulb. Enter the patient’s information if necessary.
3. Determine which program the patient is currently using and switch to another existing program (Fig. 3). Make sure to increase the amplitude until the patient can feel the stimulation. Make note of where the patient feels the stimulation. One common program configuration in the perioperative follow-up period is:
  - “C1” electrode 0 NEGATIVE electrode 3 POSITIVE
  - “C2” electrode 1 NEGATIVE electrode 3 POSITIVE
  - “C3” electrode 2 NEGATIVE electrode 0 POSITIVE
  - “C4” two electrodes set to NEGATIVE and the furthest from both set to POSITIVE
4. If stimulation cannot be achieved by changing to an existing program, it may be necessary to change the active (NEGATIVE or “−”) electrode to a different location. It is important to always give the POSITIVE electrode.
5. If altering the active electrode is still not achieving stimulation in the desired location, it may be time to modify pulse width (“msec”) or frequency (“Hz”) of the stimulation signal. This maneuver can alter where the patient feels the stimulation by recruiting different nerve bundles or nerve roots.

## Algorithm 3

### Modifying Pulse Width and Frequency to Alter Where the Patient Feels the Stimulation

**Fig. 5:**
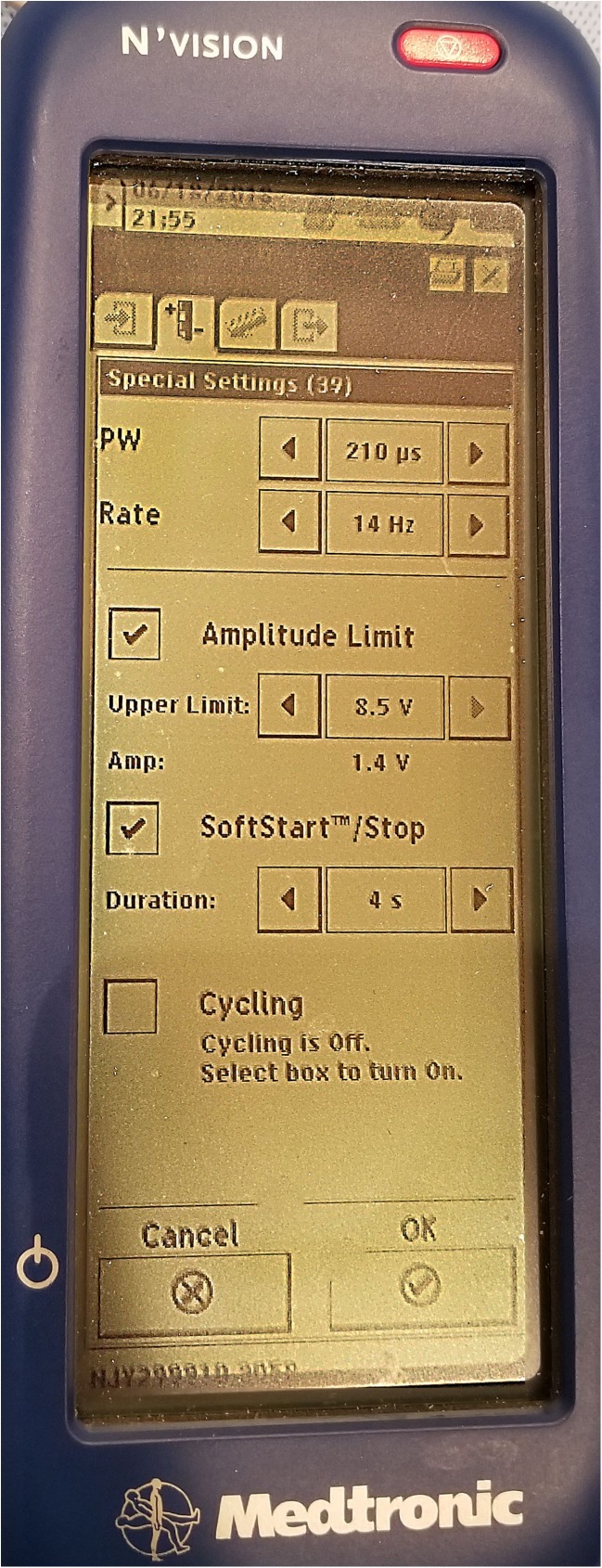
Stimulation signal modification screen. The area to the right of “PW” demonstrates the pulse width modification icon which is normally set to 220 μs but can achieve stimulation from 60 to 400 μs. The area to the right of “Rate” demonstrates the frequency modification icon, normally set from 9.7 to 12 Hz, but which can be adjusted from 9.0 up to 60 Hz. The higher frequency stimulation is associated with more intense and sometimes uncomfortable stimulation, allowing the clinician to decrease the stimulation amplitude and sometimes recruiting different nerve root bundles allowing the clinician to salvage leads that otherwise may not be stimulating in the appropriate areas. “Amplitude Limit” sets a limit on how high the patient can turn up the stimulation, normally set to the maximum 10 V. “Soft Start/Stop” allows a gentle ramping up of stimulation so that when stimulation is initiated, it is not as startling to the patient. “Cycling” allows the SNS generator to turn itself “ON” and “OFF” every 8 s (set by the clinician), for a period of time set by the clinician

1. Perform basic interrogation and basic routine for changing the program. Some have noted that the motor response is more predictive for a good long-term successful implant than the sensory response [20], but increasing the stimulation amplitude to levels sufficient to elicit a motor response outside of the operating room setting is often quite uncomfortable for the awake patient in-office, so sensory response is used in clinic. Sensation that is most desirable is in the genital area, but opinions on this differ. Many try to avoid sensory stimulation affecting the thigh or toes, although a motor toe response is a desirable response during implantation, suggesting stimulation of the S3 nerve roots. Movement of the great toe following implantation at low stimulation levels can be distracting and sometimes painful for patients.
2. Modify the active electrode as described in Algorithm 2. If unsuccessful, go to the icon for altering signal parameters (see Fig. 5).
3. Identify the icon for pulse width modification (Fig. 5). The label “PW” demonstrates the pulse width modification icon which is normally set to 220 μs but can achieve stimulation from 60 μs to 450 μs. Make note of where the stimulation is set.
4. Start with a lower pulse width by 100 μs than the current setting, ask the patient if they feel this at all, then ask if the location has changed, even a little, from the pre-modification stimulation. Then increase pulse width to 100 μs higher than the starting pulse width noted upon starting the process. If the stimulation moves to a genital location close out of the menu, SYNC the device (“P” button) to send the new program to the SNM generator. If modification of the pulse width is not adequate to achieve desired sensory response, move on to modification of frequency of stimulation.
5. Identify the icon for frequency modification (Fig. 5). The label “Rate” demonstrates the frequency modification icon, normally set from 9.7 to 14 Hz, but which can be adjusted from 2.1 up to 130 Hz. Make note of the frequency to which the current stimulation is set. The higher frequency stimulation is associated with more intense and sometimes uncomfortable stimulation, allowing the clinician to decrease the stimulation amplitude and sometimes recruiting different nerve root bundles allowing the patient wider therapeutic options.
6. Increase the frequency of stimulation by 10 Hz. Warn the patient, this may be uncomfortable. Ask if they can now feel the stimulation in a different location. Any change that is not painful or uncomfortable is a reasonable stopping point.
7. SYNC the device (“P” button) to send the new program to the SNM generator.
8. After all programming is completed, move to the 4th item on the menu bar (Fig. 3) and SYNC the iCon patient programmer with the clinician programmer. If this step is not completed, or if the patient did not come with the iCon patient programmer, the new program will remain on the SNM generator only as long as the patient’s iCon patient programmer is not used to interrogate, increase the amplitude, or in general “talk” to the SNM generator. As soon as the not-updated iCon patient programmer SYNCs with the SNM generator, it will revert all programs to what it thinks they should be.

## Algorithm 4

### Modifying Test Voltage for Lead Impedance Testing

Figure 4 demonstrates the impedance testing screen, immediately below electrode impedance label appears the “Amp (V)” label. This is set to 1.0 V by default and can be increased to increments of 0.1 V. If by 2.0 V the impedance still reads “> 4000 Ω,” then it is likely that the lead is broken.
